# Early Detection of *Monilinia laxa* in Yellow-Fleshed Peach Using a Non-Destructive E-Nose Approach

**DOI:** 10.3390/foods14183155

**Published:** 2025-09-10

**Authors:** Ana Martínez, Alejandro Hernández, Patricia Arroyo, Jesús Lozano, María de Guía Córdoba, Alberto Martín

**Affiliations:** 1 Nutrición y Bromatología, Escuela de Ingenierías Agrarias, Universidad de Extremadura, 06007 Badajoz, Spain; anamd@unex.es (A.M.); mdeguia@unex.es (M.d.G.C.); amartin@unex.es (A.M.); 2 Instituto Universitario de Investigación en Recursos Agrarios (INURA), Universidad de Extremadura, Avd. de la Investigación, 06006 Badajoz, Spain; jesuslozano@unex.es; 3 Escuela de Ingenierías Industriales, Universidad de Extremadura, 06006 Badajoz, Spain; parroyoz@unex.es

**Keywords:** E-nose, volatile profile, early decay, yellow peach, *Monillia laxa*

## Abstract

This study evaluated the performance of an electronic nose (E-nose) system for the early detection of fungal spoilage in yellow-fleshed peach (*Prunus persica* cv. ‘Carla’). Fruits were divided into two groups: one inoculated with *Monilinia laxa* and a non-inoculated control. Volatile organic compounds (VOCs) were identified and quantified via gas chromatography–mass spectrometry (GC–MS), while E-nose sensor responses were recorded at two post-inoculation stages: early and middle decay. A strong correlation was observed between E-nose biosensor signals and VOC profiles associated with fungal development. Linear discriminant analysis (LDA) models based on E-nose data successfully classified samples into three categories: healthy, early decay, and middle decay. Recognition rates exceeded 97% across all external validations, with 100% accuracy for early-stage infections. These results demonstrate the potential of E-nose technology as a rapid, non-destructive tool for monitoring peach quality during storage.

## 1. Introduction

Fungal diseases significantly affect peach fruit during pre-harvest and post-harvest stages, leading to substantial economic losses. The primary pathogens causing these diseases include *Monilinia* spp. (responsible for brown rot), *Rhizopus stolonifer*, *Botrytis cinerea*, and *Colletotrichum* spp. among others [1,2,3]. Brown rot, a critical postharvest disease, is primarily attributed to fungal pathogens within the *Monilinia* genus. Traditionally, *Monilinia fructicola* has been recognized for its prevalence in North America and Asia, while *Monilinia laxa* and *Monilinia fructigena* dominated European orchards [4,5]. However, a significant epidemiological shift has occurred since the early 21st century, marked by the emergence and rapid dissemination of *M. fructicola* across the Mediterranean basin and throughout Europe. In Spanish stone fruit orchards, recent surveys indicate the establishment of a co-occurrence pattern between the invasive *M. fructicola* and the indigenous *M. laxa*, with *M. fructigena* detections becoming increasingly rare [6]. Consequently, *M. laxa* remains a substantial concern for the commercial viability of stone fruits, particularly peaches, in Spain. Not only does brown rot thrive in conditions post-harvest, but it also correlates with internal fruit deterioration mechanisms that promote rapid decay [1,7].

Early detection of these fungal pathogens is essential to mitigate their impact on fruit production. Various non-destructive techniques, including NIR spectroscopy, ultrasonic testing, hyperspectral imaging (HSI), and E-noses, offer a robust framework for the online detection of fruit quality and spoilage. These methods not only enhance the accuracy of quality assessments but also contribute to reducing food waste and improving safety standards in the fruit supply chain. In the case of NIR spectroscopy and HSI, the application of techniques has demonstrated the potential for non-destructive, rapid, and online inspection of maturity stage, evaluation of physicochemical attributes, and detection of physical defects and contaminants in fruits [8,9]. An important advantage of near-infrared (NIR) spectroscopy and hyperspectral imaging (HSI) lies in their ability to simultaneously assess multiple quality attributes. Despite this potential, their commercial implementation for the early detection and prevention of fungal diseases during fruit storage remains limited within the industry [10,11].

E-noses have emerged as a promising technology for this purpose. By detecting volatile organic compounds (VOCs) emitted by spoilage fungi, E-noses can identify contamination at an early stage, even before visible signs appear. This capability is particularly crucial given the high susceptibility of fruits to fungal contamination during various stages of their lifecycle, including pre-harvest, post-harvest, and storage [12,13]. Several investigations have confirmed the effectiveness of electronic nose (E-nose) systems in evaluating fruit quality and identifying early signs of spoilage. For instance, ref. [14] highlighted the potential of an E-nose system combined with imaging technology to monitor the volatile compounds associated with fruit ripening, suggesting that this approach could be adapted for detecting fungal infections in stone fruits. Similarly, ref. [15] discussed the integration of machine vision with E-nose technology to evaluate the freshness of postharvest products, indicating that such systems can effectively classify and sort fruits based on their quality attributes. This dual approach could be particularly beneficial in identifying early signs of *Monilinia* infection, allowing for timely interventions.

Recent research has demonstrated the effectiveness of E-noses in identifying specific fungal decays in fruits. E-nose has been used to differentiate between fresh and moldy apples inoculated with *Penicillium expansum* and *Aspergillus niger*, showcasing the technology’s potential for rapid and early detection [16,17,18]. Similarly, ref. [19] employed E-nose technology combined with gas chromatography-mass spectrometry to classify pathogenic fungal diseases in post-harvest strawberries, further validating the applicability of E-noses in various fruit types. These studies highlight the versatility of E-noses in detecting a range of fungal pathogens, making them a valuable tool in the fruit and vegetable industry.

The integration of multivariate analysis techniques in E-nose data processing is essential for extracting meaningful insights from complex datasets generated by chemical sensor arrays. One of the foundational techniques employed in the analysis of E-nose data is Principal Component Analysis (PCA), which serves to reduce dimensionality while preserving variance in the dataset. PCA transforms the original correlated variables into a set of uncorrelated variables known as principal components, facilitating the identification of patterns within the data [20,21]. This method has been effectively utilized in various studies, such as the differentiation of Peruvian wines [21] and the characterization of olive oils [7], where it helped in elucidating the relationships between sensor responses and sample characteristics.

In addition to PCA, other multivariate statistical methods such as Linear Discriminant Analysis (LDA), Partial Least Squares Regression (PLSR), and machine learning algorithms, including Support Vector Machines (SVM) and Artificial Neural Networks (ANN), have been integrated with E-nose data to improve classification performance [22,23,24]. For instance, research on the freshness monitoring of anchovies demonstrated the effectiveness of combining PCA with LDA to distinguish between different freshness stages based on sensor data [20]. Additionally, Linear Discriminant Analysis (LDA) has been employed to classify fruit spoilage types, demonstrating high accuracy in distinguishing between healthy and spoiled samples [17,18].

The objective of this study is to investigate the volatile organic compounds (VOCs) and detect biomarkers of fungal spoilage, specifically focusing on the correlation between VOCs and the metal oxide sensors (MOX) of an E-nose. The E-nose, a portable device, will be evaluated for its application as a non-destructive technique in the early detection of *Monilinia* spp. on yellow-fleshed peach.

## 2. Materials and Methods

### 2.1. Experimental Design

*Monilinia laxa* CA1 [25], from the Culture Collection of CAMIALI group research (UEx), was selected for this study. Individual cultures were maintained on potato dextrose agar (PDA) at 25 °C for a period of ten days.

Yellow-fleshed peaches (*Prunus persica* L. cv. ‘Carla’) were sourced from a major supermarket chain during June and July 2023, corresponding to their typical harvest period in late May to early June. To preserve postharvest freshness, fruits were stored at 1 °C for a maximum of 48 h prior to inoculation. Before treatment, the surfaces of peaches were sanitized using 70% ethanol, air-dried, and mechanically wounded (3 mm width × 3 mm depth) with a sterile tip. Each fruit was then inoculated by inserting a cube of *M. laxa* mycelium (3 mm × 3 mm) into the wound site. Two experimental groups were established: (i) ML-inoculated and (ii) control (non-inoculated, sound fruit).

Inoculated samples were individually placed in transparent polyethylene containers (25 × 25 × 11 cm; width × height × depth) and incubated at 25 °C. For volatile organic compound (VOC) analysis, three samples from each group were collected at two distinct infection stages, defined by lesion diameter: early stage (15 mm) and intermediate stage (25 mm), corresponding to 2–3 and 4–5 days post-inoculation, respectively. A batch of sound peaches was included as a control. All experimental procedures were conducted in triplicate to ensure reproducibility and statistical robustness.

### 2.2. Volatile Compound Analysis

#### 2.2.1. Volatile Extraction

To capture VOCs, the samples were placed inside glass jars with plastic lids, each having a capacity of 780 cm^3^. These jars were pre-conditioned before sealing. The headspace was flushed with GC-grade air for 2 min to eliminate residual VOCs from previous samples. Volatile extractions were conducted by directly inserting the 75 µm Carboxen/polydimethylsiloxane (CAR/PDMS)HS-SPME fiber (Supelco, Bellefonte, PA, USA) through the jar lid, with VOCs being extracted at 25 °C for 40 min. Gas chromatography/mass spectrometry (GC/MS) analysis was performed as detailed in Section 2.2.2. Each analysis was conducted in triplicate, and the entire experiment was repeated three times to ensure reliability. The fibers were activated according to the conditioning guidelines prior to their initial use.

#### 2.2.2. Gas Chromatography/Mass Spectrometry (GC/MS) Analysis

Volatile organic compounds (VOCs) were identified and quantified by gas chromatography–mass spectrometry (GC–MS), following the protocol described by [26]. The analyses were performed using an Agilent 6890 gas chromatograph coupled to a 5973 mass selective detector (Agilent Technologies, Little Falls, DE, USA). Compound separation was carried out on a capillary column (HP-5MS equivalent; model 19091J-215, Agilent Technologies, Madrid, Spain) measuring 50 m in length, with an internal diameter of 0.32 mm and a film thickness of 1.05 μm, coated with 5% phenyl and 95% polydimethylsiloxane.

Gas chromatography–mass spectrometry (GC–MS) analysis was performed using helium as the carrier gas at a pressure of 9.1 psi, yielding a flow rate of 1.2 mL min^−1^ at 40 °C. The solid-phase microextraction (SPME) fiber was thermally desorbed and held in the injection port at 250 °C throughout the chromatographic run (43.50 min), operated in splitless mode. The oven temperature program began with an isothermal hold at 40 °C for 6 min, followed by a ramp to 150 °C at 4 °C min^−1^ until 33.50 min, and then increased to 250 °C at 20 °C min^−1^, maintained for an additional 5 min. The GC–MS transfer line was set at 300 °C. Mass spectrometric detection was conducted in electron impact mode at 70 eV, with a multiplier voltage of 1650 V. Data acquisition was performed at a scan rate of 1 scan s^−1^ over a mass range of *m*/*z* 40–250.

Compound identification was carried out by combining Kovats retention indices—calculated using a series of n-alkanes (R-8769, Sigma Chemical Co., St. Louis, MO, USA)—with spectral matching against the NIST/EPA/NIH mass spectral library, ensuring a match quality exceeding 90%. For additional confirmation, selected compounds were validated by comparing both retention times and mass spectra with a laboratory-compiled reference library generated from pure standards analyzed under identical chromatographic conditions. Quantification was based on total ion current (TIC) chromatograms, with peak areas integrated to estimate relative abundances.

### 2.3. E-Nose Analysis

#### 2.3.1. E-Nose System

This investigation utilized an E-nose system, engineered by the Sensory Systems research group at the University of Extremadura [27]. The E-nose comprises an array of eleven commercially available metal oxide semiconductor (MOX) sensors, affording a broad spectrum of detection capabilities. These sensors are sourced from various manufacturers, as follows: (i) Bosch BME680: Measures environmental parameters including temperature (°C), pressure (hPa), humidity (%RH) and gas resistance (Ω); (ii) Sensirion SGP30: Detects equivalent CO_2_ (eCO_2_) concentration, total volatile organic compound (TVOC) concentration, and raw resistive values associated with hydrogen and ethanol. (iii) ScioSense CCS811 and (iv) iAQ-Core: Measure eCO_2_, TVOC, and sensor resistance.

Each sensor is encapsulated within a compact module that integrates analog conditioning circuits, analog-to-digital converters, a microcontroller, communication interfaces, and a heated microplate.

#### 2.3.2. Measurement Process and Data Analysis

The measurement protocol of the E-nose system comprises two distinct operational phases: (i) Adsorption Phase (120 s): During this interval, the sensors are directly exposed to the headspace of the sample, facilitating the adsorption of volatile organic compounds (VOCs). (ii) Desorption Phase (120 s): In this phase, the sensors are exposed solely to ambient air, thereby establishing a baseline for subsequent odor discrimination. 

To derive a representative value from each measurement cycle, a baseline correction algorithm was implemented. The characteristic response was computed as the proportional difference between the baseline signal (V_ref_) and the signal recorded during exposure to volatiles (V_odor_). The baseline value was defined as the arithmetic mean of the final five data points obtained during the desorption phase, whereas the volatile value corresponded to the mean of the final five data points recorded during the adsorption phase. The final value (V_f_) was calculated using the following expression:

V_f_ = (V_ref_ − V_odor_) × 100 (1)

### 2.4. Statistical Analysis

Data analysis was conducted using SPSS 15.0 for Windows (SPSS Inc., Chicago, IL, USA). Descriptive statistics were calculated for the volatile organic compounds (VOCs) measured. Two-way analysis of variance (ANOVA) was employed to investigate the effects of inoculation and stage factors on VOC profiles. Principal component analysis (PCA) was performed on the volatile compound values to determine their discriminatory potential between the studied samples. To evaluate the performance of electronic nose (E-nose) sensors as indicators of volatile profile variations in yellow-fleshed peaches, Pearson correlation coefficients and hierarchical cluster analysis (HCA) were applied. Sample similarity was calculated using squared Euclidean distance, and clustering was performed via hierarchical agglomerative linkage between groups.

Linear discriminant analysis (LDA) was employed to classify peach batches across ripening stages. A stepwise selection algorithm based on Wilks’ lambda was used to identify the most relevant discriminant variables for both early and intermediate stages of ripening. The classification outcomes from the optimal LDA model were visualized by projecting the peach groups into the space defined by the discriminant functions (DFs).

## 3. Results and Discussion

### 3.1. Volatile Compounds

Peach fruit (*P. persica* L.) is characterized by a complex profile of volatile compounds that significantly contribute to its aroma and flavor. The volatile composition of peaches is influenced by various factors, including genetic background, ripening stages, and postharvest conditions [28,29,30]. Research has identified over 100 volatile compounds in white and yellow-fleshed peaches, which can be categorized into several sensory groups, such as green, fruity, and floral aromas. Among these, key compounds include hexanal, (E)-2-hexenal, linalool, and γ-decalactone [30,31,32]. In our study, we identified a total of 46 compounds emitted by the peach samples, which were categorized into the following chemical families: esters (33), hydrocarbons (7), alcohols (4), lactones (1), and phenol (1) (Table 1).

**Figure 1:**
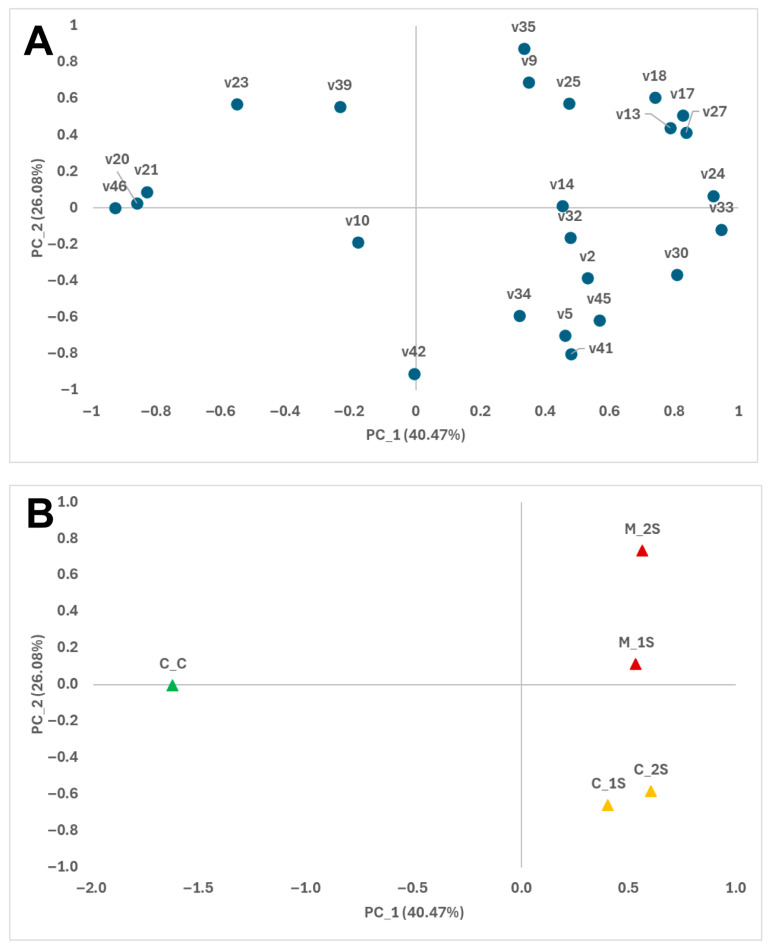
Loading plot (**A**) and score plot (**B**) derived from the principal component analysis (PCA) of volatile compounds in peach samples, displayed in the two-dimensional space defined by the first and second principal components (PC1 and PC2). Volatile compounds (code described in Table 1; ●); Control samples Day 0 (C_C; ▲); Uninoculated samples (▲); Inoculated samples (▲); Early stage (_s1), Middle stage (_s2).

Esters is the most abundant family, and the major compounds are ethyl octanoate (39.63%), ethyl (Z)-4-octenoate (12.23%), ethyl acetate (8.71%) and ethyl hexanoate (6.77%). After storage, esters tended to increase and predominate in the volatile profiles (Table 1), in concordance with the results obtained by other authors for yellow-fleshed peach cultivars [33,34]. This evolution is also evident in the principal component analysis of volatile compounds that present correlation with the MOX sensors (Figure 1, Table S1). The impact of storage on the changes in volatile emissions is captured by the first principal component, which accounts for 40% of the variability. Early-stage samples are positioned on the positive axis of the first component, correlating with several minority esters, mostly unsaturated (v13, v17, v24, v27, v30, and v33) and other compounds such as octane (v14). Conversely, high levels of several aromatic hydrocarbons, including ethylbenzene (v20), p-xylene (21), and styrene (23), are associated with the negative axis, corresponding to the unstored control samples (Figure 1; Table 1).

**Figure 2:**
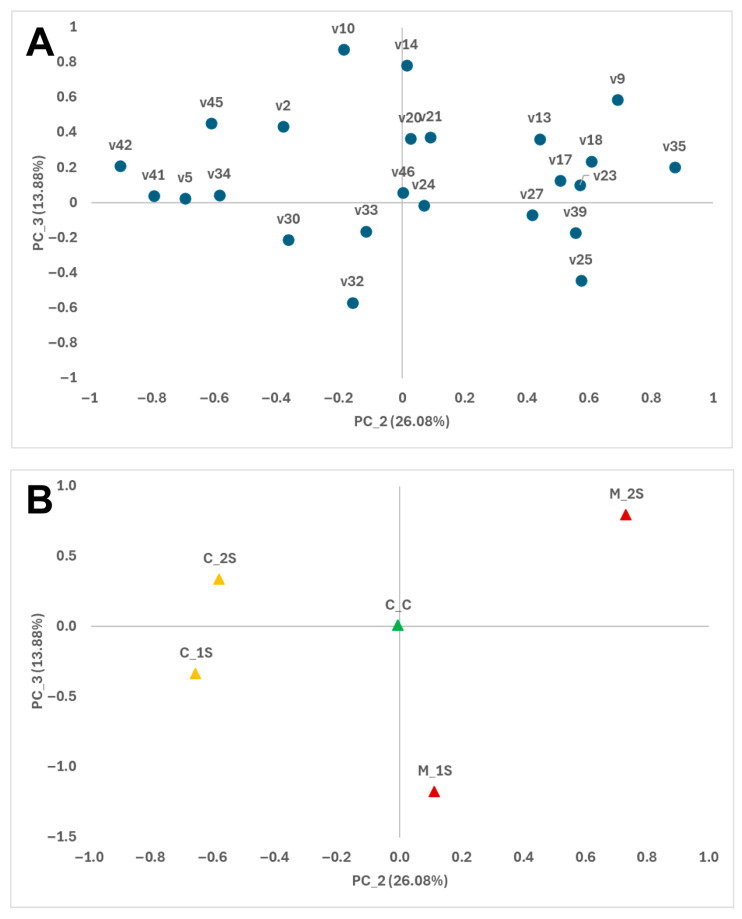
Loading plot (**A**) and score plot (**B**) derived from the principal component analysis (PCA) of volatile compounds in peach samples, displayed in the two-dimensional space defined by the second and third principal components (PC2 and PC3). Volatile compounds (code described in Table 1; ●); Control samples Day 0 (C_C; ▲); Uninoculated samples (▲); Inoculated samples (▲); Early stage (_s1), Middle stage (_s2).

Furthermore, the impact of *Monilinia* inoculation on the volatilome emitted by stored peaches is illustrated in Figure 2, which represents the plane defined by the second and third principal components. The second component, accounting for 26.08% of the variability, distinguishes sound peaches from inoculated fruits. Higher levels of compounds ‘Unidentified Ester’ (v35), ethyl 2-methylbutanoate (v18), and styrene (v23) are associated with inoculated fruits on the positive axis. In contrast, sound peaches positioned on the negative axis exhibit higher levels of ethyl octanoate (v41), ethyl 2-octenoate (v42), and n-propyl acetate (v5) (Figure 2). All mentioned compounds showed significant differences between control and inoculated peaches (Pi < 0.050; Table 1).

Regarding the third principal component, which accounts for 13.88% of the variability, compounds toluene (v10) and octane (v14) are primarily associated with inoculated peaches after 4–5 days of storage and are located on the positive axis (Figure 2). Conversely, (Z)-2-hexenyl acetate (v32) and, to a lesser extent, pentyl acetate (v25) are associated with inoculated peaches after 2–3 days of storage and are positioned on the negative axis. In conclusion, the inoculation with *M. laxa* markedly alters the volatile compound profile emitted by stored peaches. This alteration distinctly differentiates between sound and inoculated fruits, as well as between various storage durations. These findings underscore the potential application of an E-nose for the early detection of *Monilinia* during peach storage.

### 3.2. Relationship Between VOCs and Signals from E-Nose Sensors

Hierarchical Cluster Analysis (HCA) serves as a pivotal statistical method employed in the interpretation of data generated by E-noses. This technique enables researchers to categorize datasets into clusters based on the similarity of sensor responses, thereby enhancing the understanding of odorants and their properties [35,36,37].

The application of HCA in E-nose technology spans various fields, including food quality assessment and agricultural research. For instance, HCA was used to classify different fish sauce products based on their olfactory signatures, demonstrating the method’s capability to differentiate between similar yet distinct samples [38]. Additionally, HCA has been significant in agricultural applications, allowing for differentiation of fruit ripeness levels essential for quality control in food supply chains [14,39]. The classification results obtained from HCA show marked improvement with the incorporation of multiple data sources, such as combining E-nose data with other parameters for greater accuracy [40].

In our study, Table 2 illustrates the clustering of volatile organic compounds (VOCs) based on their correlations with the signals from the E-nose sensors. The analysis revealed a robust sensor response, with significant correlations identified for 24 VOCs. Specifically, Cluster 2, which comprises seven VOCs predominantly consisting of branched and unsaturated ethyl esters with 4 to 6 carbon atoms, exhibited positive correlations with the signals from sensors RBME680, ResohmCCS811, and H_2_SGP30, while showing negative correlations with TVOCCCS811 and CO_2_CCS811 (Table 2). Conversely, Cluster 4, which includes aromatic hydrocarbons and ethyl esters derived from acids with 8 carbon atoms, demonstrated an inverse response pattern. Methyl octanoate, toluene, and n-propyl acetate, categorized within Cluster 1, exhibited similar behavior, except for no correlation observed for sensors TVOCSGP30 and TVOCCCS811 (Table 2). The relationship between the aforementioned metal oxide (MOX) sensors and their response to esters has been previously described by [18]. Their research highlights the application of this E-nose device in the early detection of decay in ‘Golden Delicious’ apples caused by *P. expansum*. In the context of the volatile profile of the yellow-fleshed peach, the sensors demonstrated a low response to the VOCs grouped in Cluster 3 (Table 2).

The findings demonstrate the effectiveness of metal oxide (MOX) sensors as tools for developing discriminative models based on the relationship between MOX sensor responses and the most relevant volatile organic compounds (VOCs) emitted by yellow-fleshed peaches during postharvest storage.

### 3.3. Determination of Incipient Fungal Decay of Peaches by E-Nose During Postharvest Storage

Linear Discriminant Analysis (LDA) models were developed using E-nose sensor responses to monitor the storage of peaches from both control and inoculated batches. PCA results of volatile compounds guided the use of leave-more-out cross-validation with two groups (control and diseased fruit) to test the predictive capability of the LDA models.

**Figure 3:**
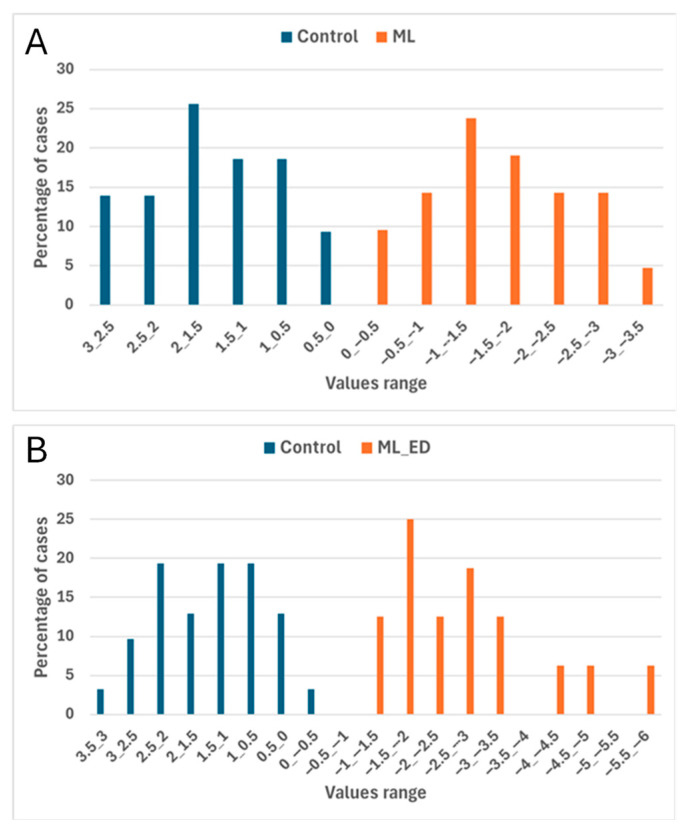
Histogram of peach samples classified into control and inoculated batches based on discriminant function (DF) scores for early-stage samples (**A**) and the entire dataset (**B**).

The LDA results for classifying sound and fungal decay fruits are summarized in Table 3, which lists the E-nose sensors (ordered by decreasing importance) selected by the stepwise algorithm for early infected and total samples and the number (and percentage) of correct classifications in both calibration and prediction phases. The LDA of E-nose data collected during peach storage confirmed the utility of certain sensors in discriminating between peach batches. In particular, sensors TVOCCCS811, CO_2_CCS811, and CO_2_SGP30 were common discriminant variables for both models (total decay fruit and incipient decay fruit), and TVOCSGP30, CO_2_iAQ, and TVOCiAQ were also important. Figure 3 illustrates the percentage distribution of early infected and total samples within the discriminant function (DF) ranges. The percentages of correctly recognized external samples were 100% and 97.2% for incipient and total decay samples, respectively (Table 3). Thus, LDA models based on E-nose sensor data effectively differentiated the sample groups.

Previous research has highlighted the potential of electronic nose (E-nose) systems combined with linear discriminant analysis (LDA) for the early detection of postharvest diseases in fruit. For example, ref. [41] applied LDA to E-nose data to identify soft rot in kiwifruit, achieving classification accuracies of 72.73% for the training set and 87.50% for the validation set. Similarly, ref. [42] utilized E-nose technology to detect latent fungal infections caused by *Alternaria* spp. in pomegranate, reaching 100% accuracy using only two metal oxide (MOX) sensors. In another study, ref. [18] evaluated the performance of an E-nose system in detecting early spoilage in ‘Golden Delicious’ apples due to *P. expansum*, reporting over 97% accuracy in distinguishing healthy from infected samples.

**Figure 4:**
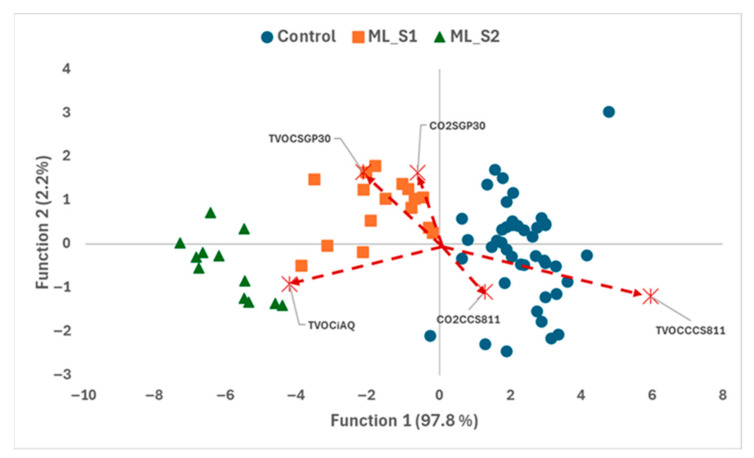
Peach samples grouped into batches: Control (uninoculated sound samples; ●), ML_S1 (inoculated sample in early stage; ■) and ML_S2 (inoculated samples in middle stage; ▲) and variable loadings projected on DF1 and DF2 plane.

In the case of peaches, several studies have demonstrated the effectiveness of E-nose systems in assessing freshness, fungal and mechanical decay, and shelf life across different cultivars [34,43,44,45]. However, most of these investigations did not examine the relationship between volatile compound profiles and sensor responses. Notably, ref. [45] demonstrated the capability of E-nose technology to discriminate among three common spoilage fungi—*Botrytis cinerea*, *Monilinia fructicola*, and *Rhizopus stolonifer*—during peach storage. In our study, the LDA model was applied to differentiate between early- and mid-stage inoculated samples, maintaining high classification accuracy using the same set of discriminant variables (Figure 4). Specifically, 97.2% of the external validation samples were correctly classified (Table 4).

## 4. Conclusions

In this paper, we discuss the utility of E-nose sensors for quality control of peach cv. ‘Carla’ during storage, confirming the high correlation between the signals of several biosensors and the aromatic profile associated with fungal alterations by *M. laxa* in this fruit. The analysis of volatile organic compounds (VOCs) revealed distinct profiles associated with fungal contamination by *M. laxa* in yellow peaches. Compounds such as ethyl 2-methylpropanoate, ethyl (E)-2-butenoate, ethyl 2-methylbutanoate, 3-methylbutyl acetate, and 1-pentene-3-ol were found in higher concentrations in inoculated samples, whereas n-propyl acetate, ethyl heptanoate, ethyl octanoate, ethyl 2-octenoate, and ethyl decanoate were more abundant in non-inoculated fruit. Overall, ester accumulation increased during storage, with compounds like diethyl carbonate and ethyl (E)-2-methyl-2-butenoate serving as potential markers of early-stage fungal infection.

Linear discriminant analysis (LDA) applied to E-nose sensor data demonstrated robust classification performance, with specific sensors—TVOCCCS811, TVOCiAQ, TVOCSGP30, CO_2_CCS811, and CO_2_SGP30—identified as key discriminant variables for detecting incipient rot. The model achieved 100% accuracy in distinguishing between healthy and infected batches.

To the best of our knowledge, this is the first report demonstrating the discrimination of early fungal alterations caused by *M. laxa* in yellow peaches using E-nose sensor signals. These findings confirm the feasibility of E-nose systems for real-time, non-destructive monitoring during postharvest storage. For successful industrial implementation, external validation of LDA models is essential to ensure reliability and interpretability. Future research should also explore advanced data processing techniques, such as artificial neural networks (ANNs), to enhance classification performance and system adaptability.

## Figures and Tables

**Table 1:** Volatile compounds identified in peach samples.

RT ^1^	CD ^2^	Volatile Compounds	ID ^3^	IK ^4^	Mean ^5^		RSD (%) ^6^	*p* Values ^7^	
					AAU	%		Ps	Pi
	***Hydrocarbons***				7820	2.56			
4.6	v10	Toluene	A	756	814	0.24	73	+	
5.7	v14	Octane	A	796	128	0.03	153	+++	
8.4	v20	Ethylbenzene	A	855	845	0.29	104	−−−	
8.7	v21	p-Xylene	A	862	4446	1.49	89	−−−	
9.7	v23	Styrene	A	883	638	0.20	64	−−	++
14.3	v28	Branched hydrocarbon	D	983	682	0.19	33		
36.2	v46	Heptadecane	A	1700	267	0.12	194	−−−	
	*Alcohols*				3301	1.05			
2.8	v3	1-pentene-3-ol	B	683	159	0.05	182		+++
3.8	v7	3-methylbutan-1-ol	A	726	2590	0.84	193		
3.9	v8	(s)-2-methylbutan-1-ol	A	730	299	0.09	235	+++	+++
4.7	v11	(z)-2-penten-1-ol	B	759	253	0.07	236	+++	+++
	*Lactones*				3859	1.07			
17	v34	γ-Hexanolactone	B	1045	3859	1.07	28		−
	*Esters*				358,843	95.16			
1.7	v1	Methyl acetate	A	522	1058	0.28	94	++	
2.1	v2	Ethyl acetate	A	608	39,442	8.71	106	+	
3.3	v4	Ethyl propionate	B	707	693	0.17	202		
3.4	v5	n-propyl acetate	B	711	3160	0.89	27	+	−−
3.5	v6	Methyl butyrate	B	715	680	0.18	332		
4.4	v9	Ethyl 2-methylpropanoate	B	748	220	0.06	187	+++	+++
4.9	v12	Isobutyl acetate	B	767	1249	0.38	101	++	+++
5.3	v13	Diethyl carbonate	B	781	952	0.24	86	+++	++
5.9	v15	Ethyl butanoate	A	802	22,211	6.11	28	++	+
6.5	v16	Butyl acetate	A	815	341	0.09	95		
7.8	v17	Ethyl (E)-2-butenoate	B	843	1854	0.47	85	+++	+++
8	v18	Ethyl 2-methylbutanoate	B	847	2111	0.54	94	+++	+++
8.2	v19	Ethyl 3-methylbutanoate	B	851	9757	2.84	38		++
9.3	v22	3-methylbutyl acetate	B	874	4671	1.36	91	++	+++
10.4	v24	Ethyl pentanoate	B	898	536	0.14	25	+++	
11	v25	Pentyl acetate	B	911	1170	0.32	103		+++
12.2	v27	Ethyl (E)-2-methyl-2-butenoate	B	937	3209	0.81	77	+++	+++
14.9	v29	Ethyl hexanoate	B	996	25,282	6.77	13	+++	−−
15.2	v30	Ethyl (Z)-3-hexenoate	B	1002	18,525	4.54	50	+++	
15.5	v31	Hexyl acetate	B	1010	917	0.24	66	++	−−
15.6	v32	(Z)-2-hexenyl acetate	B	1012	1800	0.44	97		
16.7	v33	Ethyl 2-hexenoate	B	1038	1169	0.28	65	+++	
18.4	v35	Unidentified Ester	D	1079	331	0.10	195	+++	+++
18.8	v36	Ethyl (E)-4-heptenoate	B	1088	1854	0.51	38	++	+++
19	v37	Ethyl heptanoate	B	1093	15,849	4.13	19	+++	−−−
19.6	v38	Methyl (Z)-4-octenoate	B	1115	567	0.16	46		+++
20	v39	Methyl octanoate	B	1132	1515	0.46	59		++
22.4	v40	Ethyl (Z)-4-octenoate	B	1187	46,281	12.23	19	+++	−−−
22.8	v41	Ethyl octanoate	B	1199	142,677	39.63	21	+++	−−−
24.5	v42	Ethyl 2-octenoate	B	1243	1117	0.32	50		−−−
26.2	v43	Ethyl nonanoate	B	1319	1866	0.46	47	+++	−−−
29	v44	Ethyl (E)-4-decenoate	C	1385	3057	0.71	105		−
29.5	v45	Ethyl decanoate	B	1391	2722	0.59	101	+++	−−−
	*Phenol*				608	0.19			
11.2	v26	Methoxyphenyl-oxime	C	915	608	0.19	163		+++

^1^ RT: Retention time (min). ^2^ CD: Code of volatile compound used in Figure 1 and Figure 2. ^3^ ID: Reliability of identification: A, Compound positively identified through comparison with authentic standards; B, Tentatively identified based on spectral matching with the NIST/EPA/NIH mass spectral library (match quality > 90%) and Kovats retention index; C, Tentatively identified using the NIST/EPA/NIH mass spectral library (match quality < 90%); D, Compound not identified. ^4^ KI: Kovats retention index. ^5^ AAU: Arbitrary Area Units; (%): Relative percentage. ^6^ RSD (%): Relative Standard Deviation of AAU. ^7^ Ps: *p*-values associated with the stage factor; Pi: *p*-values associated with the inoculation factor. Statistical significance is indicated by symbols: *+* for positive effects and − for negative effects. One, two, or three symbols denote significance levels of *p* < 0.1, *p* < 0.05, and *p* < 0.01, respectively.

**Table 2:** Cluster analysis of volatile compounds based on Pearson correlation between compound peak areas and MOX sensor response values.

Cluster		MOX ^1,2^										
	VOCs	M_1	M_2	M_3	M_4	M_5	M_6	M_7	M_8	M_9	M_10	M_11
1	Toluene	-			-	-	+		-			-
1	Methyl octanoate		+		-	-				+	+	-
1	n-Propyl acetate	-	+		-	-	+		-			--
2	Diethyl carbonate	++	-	--	+	+	--	--	++			++
2	Ethyl (E)-2-butenoate	++	-	--	+	+	--	--	++			+
2	Ethyl 2-methylbutanoate	++	-	--	+		--	--	++			+
2	Ethyl pentanoate	++		-	+		-	--	++			
2	Ethyl (E)-2-methyl-2-butenoate	++		-	+		--	-	++			+
2	Ethyl (Z)-3-hexenoate	+	-		++	++	--		++	-	-	++
2	Ethyl 2-hexenoate	++	--	--	++	++	--	--	++	-	-	++
3	Octane			-								
3	Ethyl acetate		-			+				-	-	
3	Pentyl acetate	+										
3	(Z)-2-Hexenyl acetate									-	-	
3	Unidentified Ester							-				
3	Ethyl decanoate				+	+						
3	Ethyl 2-methylpropanoate							-				
4	Ethylbenzene	--	++	+	--	--	++	+	--	++	++	--
4	p-Xylene	--	++	+	--	--	++	+	--	++	++	--
4	Styrene	-	+	+	--	--	++		--	++	++	--
4	γ-Hexanolactone	--	+	++	-	-	+	++	--	+	+	
4	Ethyl octanoate	--	++	++	--	--	++	++	--	++	++	--
4	Ethyl 2-octenoate	--	++	+	--	--	++	++	--	+	+	--
4	Heptadecane	--	++	++	--	--	++	++	--	++	++	--

^1^ MOX: RBME680 (1); CO_2_SGP30 (2); TVOCSGP30 (3); H_2_SGP30 (4); EtanolSGP30 (5); CO_2_CCS811 (6); TVOCCCS811 (7); ResohmCCS811 (8); CO_2_iAQ (9); TVOCiAQ (10); RiAQCore (11). ^2^ Positive (+) or negative (-) correlation at a *p* < 0.01 significance level; Positive (++) or negative (--) correlation at a *p* < 0.05 significance level).

**Table 3:** Performance in calibration and prediction of Linear Discriminant Analysis (LDA) classification applied to the peach samples grouped in two batches.

Samples	Selected Variable	Count	Peach Batches		Total
			Control	*Monilinia*	
**Early stage**	TVOCCCS811	n	31	16	47
	TVOCiAQ	Computed classes			
	TVOCSGP30	Class ^1^	31	16	47
	CO_2_CCS811	% ^2^	100	100	100
	CO_2_SGP30	Predicted classes			
		Class ^1^	31	16	47
		% ^2^	100	100	100
All	CO_2_CCS811	n	43	28	71
	CO_2_iAQ	Computed classes			
	TVOCCCS811	Class ^1^	43	26	69
	CO_2_SGP30	% ^2^	100	92.9	97.2
		Predicted classes			
		Class ^1^	43	26	69
		%^2^	100	92.9	97.2

^1^ Number of cases correctly classified. ^2^ Percentage of cases correctly classified.

**Table 4:** Calibration and prediction performance of Linear Discriminant Analysis (LDA) applied to peach samples classified as control and spoiled (early- and mid-stage infection).

Samples	Selected Variable	Count	Peach Batches			Total
			Control	*Monilinia*		
				S_1	S_2	
All	TVOCCCS811	n	43	16	12	71
	TVOCiAQ	Computed classes				
	TVOCSGP30	Class ^1^	43	15	12	70
	CO_2_CCS811	% ^2^	100	93.8	100	98.6
	CO_2_SGP30	Predicted classes				
		Class ^1^	42	15	12	69
		% ^2^	97.7	93.8	100	97.2

^1^ Number of cases correctly classified. ^2^ Percentage of cases correctly classified.

## Data Availability

The original contributions presented in this study are included in the article/Supplementary Material. Further inquiries can be directed to the corresponding author.

## Supplementary Materials

The following supporting information can be downloaded at https://www.mdpi.com/article/10.3390/foods14183155/s1, Table S1: Relative concentration (%) of volatile compounds identified in peaches.; Table S2: Pearson correlation values between their area and the responses values of the different MOX used.

## Abbreviations

The following abbreviations are used in this manuscript:

GC/MS	Gas Chromatography/Mass Spectrometry
LDA	Linear Discriminant Analysis
PCA	Principal Component Analysis
NIR	Near-Infrared Spectroscopy
HSI	Hyperspectral imaging
VOCs	Volatile organic compounds
PLSR	Partial Least Squares Regression
SVM	Support Vector Machines
ANN	Artificial Neural Networks
MOX	Metal Oxide Sensors
E-nose	Electronic nose
HCA	Hierarchical Cluster Analysis
HS-SPME	Headspace Solid-Phase Microextraction
