# Supplementary material for: Early Detection of Monilinia laxa in Yellow-Fleshed Peach Using a Non-Destructive E-Nose Approach
Source: Foods. 2025 Sep 10;14(18):3155. doi: 10.3390/foods14183155 (PMC12469033; doi:10.3390/foods14183155)
Supplement: Supplementary file 1 [file foods-14-03155-s001.zip › Table S1.pdf]

Table S1. Relative concentration (%) of volatile compounds identified in peaches.

Volatile compound (ID)	Control (day 0)			No inoculated						*Monilia Laxa*						P Values	
				2-3 days			4-5 days			2-3 days			4-5 days				
	Mean		SD	Mean		SD	Mean		SD	Mean		SD	Mean		SD	Ps	Pi
**Hydrocarbons**																	
Toluene	0.45	±	0.10	0.18	±	0.02	0.24	±	0.02	0.00	±	0.00	0.28	±	0.25	+	
Octane	0.00	±	0.00	0.00	±	0.00	0.09	±	0.04	0.00	±	0.00	0.09	±	0.01	+++	
Ethylbenzene	0.76	±	0.22	0.12	±	0.03	0.13	±	0.02	0.07	±	0.00	0.23	±	0.08	---	
p-Xylene	3.52	±	0.96	0.68	±	0.05	0.76	±	0.03	0.47	±	0.03	1.40	±	0.29	---	
Styrene	0.38	±	0.11	0.10	±	0.00	0.10	±	0.01	0.17	±	0.01	0.24	±	0.08	--	++
Heptane. 2.2.4.6.6-pentamethyl	0.24	±	0.05	0.20	±	0.04	0.11	±	0.04	0.14	±	0.00	0.25	±	0.02		
Heptadecane	0.47	±	0.19	0.00	±	0.00	0.00	±	0.00	0.00	±	0.00	0.00	±	0.00	---	
**Alcohols**																	
1-Penten-3-ol	0.00	±	0.00	0.00	±	0.00	0.00	±	0.00	0.09	±	0.13	0.18	±	0.03		+++
1-Butanol. 3-methyl	1.76	±	3.05	0.06	±	0.10	0.06	±	0.08	0.10	±	0.14	2.16	±	0.25		
1-Butanol. 2-methyl-(s)	0.00	±	0.00	0.00	±	0.00	0.00	±	0.00	0.00	±	0.00	0.52	±	0.06	+++	+++
2-Penten-1-ol (z)	0.00	±	0.00	0.00	±	0.00	0.00	±	0.00	0.00	±	0.00	0.44	±	0.08	+++	+++
**Ketone**																	
2(3H)-Furanone. 5-ethyldihydro	1.41	±	0.04	0.90	±	0.21	1.14	±	0.41	0.91	±	0.02	0.88	±	0.36		-
**Esters**																	
Acetic acid. methyl ester	0.21	±	0.36	0.15	±	0.20	0.33	±	0.02	0.15	±	0.04	0.67	±	0.19	++	
Ethyl acetate	0.11	±	0.19	13.06	±	####	16.47	±	0.59	6.52	±	9.22	9.51	±	####	+	
Propanoic acid. ethyl ester	0.00	±	0.00	0.39	±	0.68	0.00	±	0.00	0.13	±	0.18	0.30	±	0.02		
n-Propyl acetate	1.19	±	0.17	0.87	±	0.17	0.77	±	0.17	0.74	±	0.28	0.72	±	0.11	+	--
Butanoic acid. methyl ester	0.00	±	0.00	0.00	±	0.00	0.00	±	0.00	0.00	±	0.00	1.08	±	1.41		
Propanoic acid. 2-methyl-.ethyl ester	0.00	±	0.00	0.02	±	0.03	0.03	±	0.05	0.00	±	0.00	0.29	±	0.01	+++	+++
Isobutyl acetate	0.42	±	0.04	0.06	±	0.06	0.05	±	0.08	0.44	±	0.19	1.09	±	0.23	++	+++
Diethyl carbonate	0.00	±	0.00	0.24	±	0.02	0.21	±	0.09	0.26	±	0.12	0.60	±	0.01	+++	++

Volatile compound (ID)	Control (day 0)			No inoculated						*Monilia Laxa*						P Values	
				2-3 days			4-5 days			2-3 days			4-5 days				
	Mean		SD	Mean		SD	Mean		SD	Mean		SD	Mean		SD	Ps	Pi
Butanoic acid. ethyl ester	5.76	±	0.52	5.82	±	1.07	3.85	±	0.13	6.63	±	1.67	8.81	±	0.68	++	+
Acetic acid. butyl ester	0.06	±	0.10	0.03	±	0.06	0.11	±	0.02	0.19	±	0.00	0.08	±	0.12		
2-Butenoic acid. ethyl ester. e-	0.00	±	0.00	0.45	±	0.06	0.35	±	0.10	0.62	±	0.13	1.14	±	0.26	+++	+++
Butanoic acid. 2-methyl-.ethyl ester	0.00	±	0.00	0.43	±	0.05	0.38	±	0.07	0.69	±	0.07	1.48	±	0.02	+++	+++
Butanoic acid. 3-methyl-.ethyl ester	3.53	±	1.08	2.26	±	0.99	1.51	±	0.34	3.14	±	0.33	3.71	±	0.57		++
1-Butanol. 3.methyl-.acetate	1.05	±	0.17	0.54	±	0.10	0.39	±	0.09	1.67	±	0.15	3.69	±	1.20	++	+++
Pentanoic acid. ethyl ester	0.11	±	0.02	0.14	±	0.01	0.12	±	0.04	0.17	±	0.00	0.19	±	0.03	+++	
Acetic acid. pentyl ester	0.11	±	0.03	0.12	±	0.09	0.09	±	0.03	0.84	±	0.26	0.61	±	0.01		+++
ethyl tiglate	0.05	±	0.02	0.63	±	0.22	0.71	±	0.00	1.51	±	0.33	1.59	±	0.15	+++	+++
Hexanoic acid. ethyl ester	6.82	±	0.21	6.93	±	0.85	6.41	±	0.57	7.74	±	1.20	5.85	±	0.84	+++	--
3-Hexenoic acid. ethyl ester (Z)	1.63	±	1.33	5.79	±	1.52	5.37	±	1.75	5.76	±	2.39	4.99	±	2.23	+++	
Acetic acid. hexyl ester	0.28	±	0.24	0.29	±	0.06	0.31	±	0.04	0.26	±	0.09	0.00	±	0.00	++	--
2-Hexen-1-ol. acetate (Z)	0.12	±	0.11	0.63	±	0.60	0.25	±	0.28	0.89	±	0.43	0.35	±	0.27		
2-Hexenoic acid. ethyl ester	0.00	±	0.00	0.35	±	0.10	0.32	±	0.02	0.45	±	0.06	0.38	±	0.09	+++	
1.6-Dioxaspiro[4.4]nonane.2-ethyl	0.00	±	0.00	0.00	±	0.00	0.00	±	0.00	0.10	±	0.00	0.47	±	0.17	+++	+++
4-Heptenoic acid. ethyl ester (E)	0.42	±	0.02	0.43	±	0.07	0.32	±	0.07	0.68	±	0.03	0.79	±	0.27	++	+++
Heptanoic acid. ethyl ester	3.54	±	0.37	4.71	±	0.87	4.41	±	0.40	4.76	±	0.26	3.21	±	0.48	+++	---
4-Octenoic acid. methyl ester	0.20	±	0.01	0.09	±	0.01	0.09	±	0.03	0.20	±	0.04	0.26	±	0.07		+++
Octanoic acid. methyl ester	0.80	±	0.20	0.19	±	0.01	0.23	±	0.08	0.50	±	0.08	0.58	±	0.14		++
4-Octenoic acid. ethyl ester	11.26	±	1.17	13.94	±	2.95	11.77	±	0.34	14.07	±	1.74	9.77	±	1.89	+++	---
Octanoic acid. ethyl ester	51.83	±	4.02	37.10	±	4.11	37.87	±	0.94	36.82	±	1.45	29.68	±	4.10	+++	---
2-Octenoic acid. ethyl ester	0.54	±	0.17	0.28	±	0.04	0.34	±	0.07	0.23	±	0.03	0.14	±	0.01		---
Nonanoic acid. ethyl ester	0.30	±	0.06	0.56	±	0.01	0.80	±	0.26	0.34	±	0.01	0.30	±	0.02	+++	---
Ethyl trans-4-decenoate	0.52	±	0.24	0.54	±	0.30	1.83	±	1.57	0.49	±	0.40	0.32	±	0.00		-
Decanoic acid. ethyl ester	0.00	±	0.00	0.70	±	0.03	1.69	±	0.49	0.37	±	0.04	0.42	±	0.04	+++	---
**Phenol**																	

Volatile compound (ID)	Control (day 0)			No inoculated						*Monilia Laxa*						P Values	
				2-3 days			4-5 days			2-3 days			4-5 days				
	Mean		SD	Mean		SD	Mean		SD	Mean		SD	Mean		SD	Ps	Pi
Oxime-. methoxy-phenyl	0.14	±	0.24	0.00	±	0.00	0.00	±	0.00	0.69	±	0.33	0.21	±	0.30		+++

[1]Ps: P values of stage factor; Pi: P values of inoculation factor. The significance of the effects is indicated by + (positive effect) or - (negative effect). One. two. and three symbols correspond to p-values less than 0.1. 0.05. and 0.01. respectively.
