# Supplementary material for: Early Detection of Monilinia laxa in Yellow-Fleshed Peach Using a Non-Destructive E-Nose Approach
Source: Foods. 2025 Sep 10;14(18):3155. doi: 10.3390/foods14183155 (PMC12469033; doi:10.3390/foods14183155)
Supplement: Supplementary file 1 [file foods-14-03155-s001.zip › Table S2.pdf]

Table S2. Pearson correlation values between their area and the responses values of the different MOX used.

| Volatile compounds[1] | M1[2] | M2 | M3 | M4 | M5 | M6 | M7 | M8 | M9 | M10 | M11 |
|---|---|---|---|---|---|---|---|---|---|---|---|
| **v1** | 0.114 | -0.328 | -0.351 | 0.122 | 0.053 | -0.316 | -0.369 | 0.259 | -0.131 | -0.131 | 0.089 |
| **v2** | 0.406 | -0.618*[3] | -0.481 | 0.574 | 0.587* | -0.501 | -0.411 | 0.504 | -0.592* | -0.592* | 0.573 |
| **v3** | 0.401 | -0.245 | -0.333 | 0.262 | 0.178 | -0.435 | -0.432 | 0.408 | -0.098 | -0.098 | 0.384 |
| **v4** | 0.351 | -0.381 | -0.193 | 0.260 | 0.257 | -0.205 | -0.304 | 0.230 | -0.303 | -0.303 | 0.252 |
| **v5** | -0.611* | 0.649* | 0.533 | -0.678* | -0.616* | 0.704* | 0.543 | -0.661* | 0.501 | 0.501 | -0.760** |
| **v6** | 0.388 | -0.314 | -0.378 | 0.242 | 0.205 | -0.349 | -0.428 | 0.371 | -0.214 | -0.214 | 0.295 |
| **v7** | -0.085 | 0.214 | 0.135 | -0.214 | -0.210 | 0.160 | 0.077 | -0.124 | 0.274 | 0.274 | -0.138 |
| **v8** | 0.467 | -0.403 | -0.470 | 0.306 | 0.241 | -0.471 | -0.558 | 0.468 | -0.207 | -0.207 | 0.375 |
| **v9** | 0.519 | -0.496 | -0.562 | 0.378 | 0.319 | -0.521 | -0.630* | 0.519 | -0.289 | -0.289 | 0.423 |
| **v10** | -0.611* | 0.494 | 0.443 | -0.648* | -0.625* | 0.614* | 0.470 | -0.593* | 0.521 | 0.521 | -0.596* |
| **v11** | 0.444 | -0.391 | -0.451 | 0.295 | 0.227 | -0.462 | -0.543 | 0.451 | -0.181 | -0.181 | 0.363 |
| **v12** | 0.238 | -0.104 | -0.208 | 0.005 | -0.060 | -0.191 | -0.352 | 0.197 | 0.058 | 0.058 | 0.063 |
| **v13** | 0.777** | -0.696* | -0.742** | 0.690* | 0.626* | -0.809** | -0.793** | 0.792** | -0.551 | -0.551 | 0.711** |
| **v14** | 0.387 | -0.517 | -0.632* | 0.449 | 0.388 | -0.528 | -0.520 | 0.485 | -0.283 | -0.283 | 0.519 |
| **v15** | 0.452 | -0.189 | -0.290 | 0.170 | 0.126 | -0.317 | -0.458 | 0.343 | -0.133 | -0.133 | 0.144 |
| **v16** | 0.132 | -0.184 | -0.250 | 0.126 | 0.068 | -0.245 | -0.239 | 0.203 | -0.170 | -0.170 | 0.050 |
| **v17** | 0.778** | -0.673* | -0.715** | 0.680* | 0.608* | -0.812** | -0.790** | 0.788** | -0.528 | -0.528 | 0.689* |
| **v18** | 0.764** | -0.657* | -0.714** | 0.637* | 0.560 | -0.784** | -0.793** | 0.767** | -0.493 | -0.493 | 0.660* |
| **v19** | -0.094 | 0.445 | 0.266 | -0.352 | -0.369 | 0.265 | 0.144 | -0.228 | 0.425 | 0.425 | -0.234 |
| **v20** | -0.841** | 0.774** | 0.706* | -0.922** | -0.901** | 0.856** | 0.677* | -0.840** | 0.745** | 0.745** | -0.913** |
| **v21** | -0.828** | 0.773** | 0.697* | -0.919** | -0.906** | 0.838** | 0.662* | -0.830** | 0.771** | 0.771** | -0.905** |
| **v22** | 0.394 | -0.234 | -0.345 | 0.179 | 0.108 | -0.364 | -0.478 | 0.359 | -0.071 | -0.071 | 0.231 |
| **v23** | -0.693* | 0.697* | 0.584* | -0.846** | -0.858** | 0.711** | 0.504 | -0.709** | 0.713** | 0.713** | -0.836** |
| **v24** | 0.786** | -0.558 | -0.686* | 0.602* | 0.561 | -0.701* | -0.778** | 0.717** | -0.525 | -0.525 | 0.522 |
| **v25** | 0.580* | -0.315 | -0.428 | 0.354 | 0.277 | -0.489 | -0.551 | 0.481 | -0.252 | -0.252 | 0.330 |
| **v26** | 0.127 | 0.124 | 0.021 | 0.011 | -0.034 | -0.078 | -0.068 | 0.070 | 0.093 | 0.093 | 0.098 |

| Volatile compounds[1] | M1[2] | M2 | M3 | M4 | M5 | M6 | M7 | M8 | M9 | M10 | M11 |
|---|---|---|---|---|---|---|---|---|---|---|---|
| **v27** | 0.746** | -0.568 | -0.633* | 0.640* | 0.545 | -0.766** | -0.696* | 0.722** | -0.430 | -0.430 | 0.685* |
| **v28** | -0.237 | 0.206 | 0.153 | -0.388 | -0.345 | 0.274 | 0.032 | -0.213 | 0.123 | 0.123 | -0.514 |
| **v29** | 0.035 | 0.184 | 0.078 | -0.040 | -0.013 | 0.087 | 0.068 | -0.070 | 0.001 | 0.001 | -0.196 |
| **v30** | 0.686* | -0.656* | -0.555 | 0.784** | 0.762** | -0.744** | -0.547 | 0.716** | -0.620* | -0.620* | 0.755** |
| **v31** | -0.176 | 0.222 | 0.270 | -0.029 | 0.059 | 0.237 | 0.352 | -0.186 | 0.035 | 0.035 | 0.002 |
| **v32** | 0.466 | -0.450 | -0.359 | 0.508 | 0.533 | -0.497 | -0.430 | 0.523 | -0.580* | -0.580* | 0.362 |
| **v33** | 0.847** | -0.737** | -0.723** | 0.839** | 0.779** | -0.865** | -0.732** | 0.823** | -0.665* | -0.665* | 0.785** |
| **v34** | -0.802** | 0.679* | 0.722** | -0.686* | -0.675* | 0.691* | 0.751** | -0.717** | 0.706* | 0.706* | -0.560 |
| **v35** | 0.490 | -0.396 | -0.469 | 0.325 | 0.246 | -0.502 | -0.579* | 0.484 | -0.195 | -0.195 | 0.380 |
| **v36** | 0.466 | -0.222 | -0.339 | 0.234 | 0.164 | -0.392 | -0.485 | 0.386 | -0.126 | -0.126 | 0.247 |
| **v37** | 0.300 | -0.162 | -0.150 | 0.367 | 0.382 | -0.239 | -0.083 | 0.222 | -0.268 | -0.268 | 0.293 |
| **v38** | 0.011 | 0.170 | 0.050 | -0.234 | -0.298 | 0.051 | -0.102 | -0.052 | 0.293 | 0.293 | -0.176 |
| **v39** | -0.474 | 0.621* | 0.500 | -0.669* | -0.682* | 0.564 | 0.396 | -0.540 | 0.654* | 0.654* | -0.589* |
| **v40** | 0.185 | 0.018 | 0.062 | 0.179 | 0.191 | -0.066 | 0.060 | 0.057 | -0.092 | -0.092 | 0.131 |
| **v41** | -0.903** | 0.884** | 0.816** | -0.908** | -0.869** | 0.930** | 0.844** | -0.926** | 0.775** | 0.775** | -0.921** |
| **v42** | -0.844** | 0.737** | 0.661* | -0.806** | -0.758** | 0.835** | 0.725** | -0.826** | 0.619* | 0.619* | -0.845** |
| **v43** | 0.160 | -0.407 | -0.404 | 0.425 | 0.454 | -0.310 | -0.191 | 0.279 | -0.424 | -0.424 | 0.329 |
| **v44** | -0.165 | -0.092 | 0.094 | 0.100 | 0.057 | -0.043 | 0.215 | -0.044 | 0.091 | 0.091 | 0.238 |
| **v45** | 0.343 | -0.563 | -0.561 | 0.599* | 0.592* | -0.512 | -0.353 | 0.462 | -0.485 | -0.484 | 0.575 |
| **v46** | -0.858** | 0.820** | 0.755** | -0.933** | -0.906** | 0.888** | 0.730** | -0.872** | 0.769** | 0.769** | -0.944** |

[1]Code asigned in table 1.

[2]MOX: RBME680 (1); CO2SGP30 (2); TVOCSGP30 (3); H2SGP30 (4); EtanolSGP30 (5); CO2CCS811 (6); TVOCCCS811 (7); ResohmCCS811 (8); CO2iAQ (9); TVOCiAQ (10); RiAQCore (11).

[3]P<0.01. ***;P<0.05. **
